# Autonomy among physically frail older people in nursing home settings: a study protocol for an intervention study

**DOI:** 10.1186/1471-2318-8-32

**Published:** 2008-12-01

**Authors:** Mette Andresen, Lis Puggaard

**Affiliations:** 1 University of Southern Denmark, Faculty of Health Sciences, Institute of Exercise and Biomechanics, Denmark; 2 COWI A/S, Denmark

## Abstract

**Background:**

Experiencing autonomy is recognised to promote health and well-being for all age groups. Perceived lack of control has been found to be detrimental to physical and mental health. There is a lack of evidence-based knowledge elucidating how frail older people in nursing home settings themselves perceive autonomy in daily life. Further, there are no studies on the extent to which this perception can be influenced positively by participating in an individually tailored programme based on residents' own wishes for daily activities.

**Methods and design:**

A total of 9 nursing homes and 55 participants aged 65 years or older were included in the study. All the participants were restricted in performing at least one P-ADL activity unassisted and had a Mini Mental State Examination-score above 16. Perceived autonomy was measured at baseline, after 12 weeks and after 24 weeks by The Autonomy Sub-dimension in the Measure of Actualisation of Potential test. Programmes were based on participants' individual assessment of their most important daily activities. Staff at all nursing homes who usually organize physical training, social or creative activities carried out individually tailored programmes using their usual methods and equipment. Participants in each nursing home were divided by lot into either a control group or an intervention group. The control groups received their usual care and treatment.

**Discussion:**

This study is designed to assess the status of perceived autonomy at baseline and to provide information about the effectiveness of individually tailored programmes according to perceptions of autonomy registered in institutionalised physically frail older people. This will add knowledge to assist response to present and future challenges in relation to health promotion initiatives for this group.

**Trial registration number:**

NCT00783055

## Background

The development of elderly care services is of prime concern across Europe due to demographic trends which show an increasing elderly population [1]. The Danish Technology Council claims that in the future the elderly population will be polarised into a large group of healthy elderly and a large group of frail elderly who due to medical improvements may live with chronic diseases in more years than previously observed [2]. Therefore health promotion strategies in this group are of prime concern [3]. Only few studies of the institutionalised physically frail elderly exist and this fact makes it difficult to meet the increasing economical and practical challenges of the future [1-4].

Perceived lack of control has been found to be injurious to older peoples' physical and mental health, and when despite their frailty older people experience autonomy they become more alert both mentally and physically and their self-rated well-being improves [5-8]. Due to the limited number of studies concerning older people's perceptions of autonomy there is an evident need for more knowledge in order for politicians and planners to target the efforts to improve the documentation for health promotion strategies in this group.

### Autonomy in nursing home settings

When older people are exposed to changes such as moving into a nursing home their physical and psychological problems [5,9] and feelings of being a burden, loss of control and helplessness increase [10,11]. Furthermore, making choices and decisions in daily life risk becoming a lost ability and opportunity [11-14]. Giving up valued activities is found to threaten personal identity and indicate near-future physical decline [15]. Therefore, it is an important task for staff to support residents in making choices about engaging and re-engaging in meaningful familiar and valued activities [16]. Sensitivity towards the activity choices of the residents' is constantly at risk of receding into the background due to time schedules and the organisation of work [12,14].

### Specifying the definition of autonomy

The concept of autonomy is often used interchangeably with the experience of having choices and being in control. Perceived lack of control has been found to be detrimental to physical and mental health [6], and, when older people have feelings of control of their own activities their health and well-being are influenced positively [5-8]. Research supports the findings that older people who experience having a good life also experience being autonomous [17].

It is generally acknowledged that frail older people in nursing home settings to some degree are restricted in the execution of their choices and wishes. This calls for a more specified definition of the concept of autonomy that takes frailty and thereby dependency into consideration. According to Agich 'There is no opposition to dependency – not when one relies on others in a way that is consistent with one's sense of self-worth and identity' [18]. A number of researchers in gerontology and geriatrics have proposed focusing on the importance of making autonomous decisions irrespective of frailty, dependency and restrictions of action [4,5,17,19].

### The importance of perceiving oneself as autonomous

The sense of control over their activities exerts a positive influence on older adults' well-being [20,30] even when they are dependent on assistance with ADL (Activities of Daily Living) [5,6,13]. Furthermore, alertness and participation in daily activities increase [6,12,13] and the use of their own skills and resources strengthens the experience of self-efficacy [19,20]. This "spin-off" can be supported by the fact that preserving and strengthening physical resources have a direct impact on self-rated well-being [21-27].

Research demonstrates that it is possible to experience autonomy while being dependent on assistance, and that older people's perception of independence changes with the process of functional decline. It is, therefore, not only their actual performance but also their ability to make meaningful choices and decisions that are of importance [28]. However, there are no studies focusing on the possibility of exerting positive influence on perceived autonomy among older people in nursing home settings through individualised interventions focusing on residents' own wishes for activity in daily life.

The aim of the present study is to assess the short-term (0–12 weeks) and long-term (12–24 weeks) changes of an individually tailored programme based on residents' own wishes for activity on their perceived autonomy.

## Methods and design

The study was approved by The Regional Scientific Ethical Committee in Denmark No. 2004-1-52 and The Danish Data Protection Agency.

This study is the Danish part of a Nordic multi-centre study, where the aim is to describe the impact of individually tailored programmes in nursing home settings on residents' physical functioning, dependence in ADL and self-rated well-being. The multi-centre study includes three Nordic countries and is initiated and designed by a group of Swedish researchers.

A number of physical and mental tests are carried out and information on age, diseases, medicine, aids and length of staying at the nursing home is registered. In the Danish part of the study, a test has been added in order to measure perceived autonomy before and after the intervention period. This paper concentrates only on the assessment of the status of perceived autonomy and the effect of individually tailored programmes on perceived autonomy among physically frail older people in nursing home settings. The individual programmes are based on the participants' individual wishes according to the specific daily activities they wish to improve, to preserve and/or to revive.

**Figure 1:**
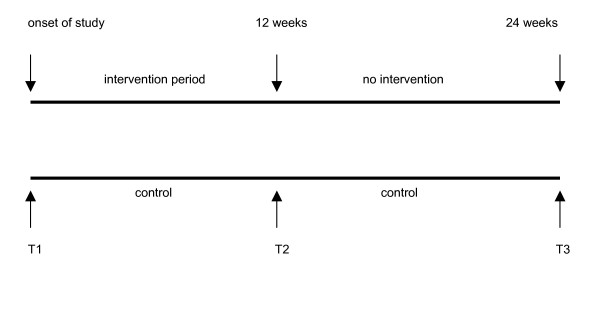
Flow chart of study design.

The intervention group participated in a 12 – week programme, whereas the control group received their usual care and treatment. After a 6 month period they were offered an individual programme according to their wishes for activities (Figure 1).

### Recruitment of nursing homes

Some 105 nursing homes built or renovated to fulfil the Danish law (On the basis of The Danish Housing Act §54, The Danish Ministry of Social affairs in cooperation with e.g. The Inspection of Work in 1997 drew up rules for the physical environment in terms of accessibility for both residents and staff) received an invitation letter informing about the project. The nursing homes were situated in a large geographical area of Denmark including rural areas, larger cities and the capital of Denmark. Invitations were sent in four rounds during a one and a half-year period from February 2005 to June 2006. A total of 20 nursing homes responded and of these 9 wanted to participate. The 9 nursing homes represent both small and larger nursing homes and are geographically placed in both the countryside and in cities.

Residents, relatives and staff from all involved nursing homes attended information meetings. Local politicians were also present at some of the meetings. Information about participation, duration of the study and clarification of the staff's roles were on the agenda and if necessary the meetings were repeated in order to ensure that everybody was informed.

### Recruitment of participants

Physically frail elderly nursing home residents were recruited directly at the above mentioned information meetings or at a visit conducted by a well-known staff member in their home. The staff at each nursing home was responsible for informing participants face to face and for collecting the signed informed consent. Participants were informed about randomisation to either an intervention or a control group.

#### Inclusion criteria

Aged 65 years or older, participants with all kinds of diseases leading to physical frailty ensuring an unselected case-mix, dependence on daily assistance in minimum one P-ADL activity (Primary ADL; e.g. eating, dressing and going to the toilet), able to understand verbal instructions, willing to participate, expected to live in the nursing home during the intervention period. Both men and women.

#### Exclusion criteria

Terminal stages of disease, MMSE-score below 16 (Mini Mental State Examination).

### Randomisation and blinding

Participants were divided by lot into either a control group or an intervention group.

The randomisation was stratified according to sex.

The intervention groups participated in individually tailored 12 – week programmes, whereas the control groups received usual care and treatment.

Participants' identity and group allocation were only identifiable for the researcher.

Blinded research assistants did the testing. Previous training sessions for testers and the use of scripts during the testing had to ensure that all participants received the same instructions.

### Description of cohort and dropout

A total of 55 older people were included according to the autonomy study of which 50 participated.

A number of measures were collected for the purpose of characterising the total cohort at baseline, in terms of age, sex, length of stay in the nursing home, most frequent diseases, most frequent medicine prescribed and walking/mobility aids (Table 1 and Table 2).

**Table 1:** Baseline characteristics of age, sex and length of stay in the nursing home divided by group.

**Intervention group**	**Control group**
Women n = 19	Women n = 16
Men n = 9	Men n = 6
Overall n = 28	Overall n = 22

**Age/yr**	**Age/yr**
Mean 84,4	Mean 83,5
Min 65	Min 66
Max 97	Max 96

**Length of stay in the nursing home/months**	**Length of stay in the nursing home/months**
Mean 32	Mean 28
Min 12	Min 7
Max 96	Max 72

**Table 2:** Baseline characteristics of most frequent diseases, most frequent medicine prescribed and mobility/walking aids of the total group.

**Most frequent diseases**	**Most frequent medicine**	**Walking/mobility aids;**
arthritis (OA, RA), hemiplegic limbs, cardiovascular problems and/or COPD	analgesics and antidepressants	3/4 of the participants use cane or rollator and 1/4 are completely wheelchair – bound

Five participants dropped out: three from the control group and two from the intervention group. Typical reasons for drop – out were withdrawal due to either aggravation of disease or death.

### General description of residents, the environment and daily life in Danish nursing homes

Descriptions of Danish nursing home residents are scarce and therefore the following characteristics are based on the one existing and recent study by Beck et al [29] which characterise 441 residents from 11 different nursing homes in Denmark in 2004. The study by Beck et al show that 50% of the residents are 85 years and above, 20% are men and more than 50% are suffering from reduced cognitive function, between 30% and 50% were totally dependent in performing P-ADL and more than 50% were medicated with analgetics on a daily basis [29].

In general, the staff comprises of nurses, auxiliary nurses, cleaning staff, occupational therapists, physiotherapists and volunteers.

Typical of the physical environment *inside *is that a reception area leads to corridors with front doors to residents' individual flats. Further, a corridor leads to common areas such as dining rooms, café, training facilities etc. Residents rent their own individual flat with a bathroom, a kitchen and a living room/bedroom. The *outside *environment usually has a common garden area with benches and flowers.

Daily life is characterised by schedules for most activities such as mealtimes, bedtimes, physical training, social or creative activities.

Nursing homes have to list their moral values – often on the nursing home's own website – and the list frequently includes *preserving residents' autonomy*.

### Specific characteristics of the 9 nursing homes

Although there were some differences, all 9 nursing homes included in this study complied with the earlier mentioned regulations about housing for the frail and old. Among the differences was for example the fact that some only consisted of a ground level while others had more floors. Some were less accessible regarding distance, e.g. to the front door, garden, training facilities and dining room.

In a few nursing homes occupational therapists and physiotherapists were employed by the local community council to serve more than one nursing home and therefore not able to be present in the nursing home on a daily basis.

### Testing perceived autonomy

Perceived autonomy was measured at T1, T2 and T3 by using *The Autonomy Sub-dimension *in the MAP test (The Measure of Actualization of Potential) [30-33]. The MAP test generally measures how older people perceive their possibilities for self-determination in daily life. Three psychologists and one occupational therapist, all from the Geriatric Institute at Sherbrooke University in Canada developed the test. Validity and reliability were tested and found high according to The Quebec Longitudinal Study on Aging [30-33].

With permission from the Canadian authors, The Autonomy Subscale was validated to be used in Denmark by the researcher under statistical supervision in 2005.

The Autonomy Sub-dimension can be used separately and represents a subjective measure which elucidates perceived autonomy. Six items are scored on a 5 – point Likert type scale and the test is performed as a paper and pencil test where the tester and the participant are sitting at the same side of the table. The tester reads the text and the participant chooses the response. Each sentence is read out loud in order to avoid misunderstandings.

The mean of all six scores forms the result and is categorised as either: *low, average *or *high degree of autonomy*.

### Training sessions for testers

The testing procedure involved a training programme concerning testing procedures in order to assure a high inter-rater reliability. The training programme ran over a three – week period and involved a thorough verbal and written presentation of test material and a manual, exercises, feed-back and de-briefing.

### Intervention

Staff at each nursing home who usually organizes physical training, social or creative activities carried out the individual programmes for the intervention groups utilizing their usual methods and equipment. The individual programme was planned together with the participant. Furthermore, the staff registered the participant's activities in terms of: *type, with whom, where, duration per time and frequency per day/week*.

### Statistical analysis

The statistical analytical program SPSS version 15.0 and 16.0 will be used for registration, analysis and presentation of data.

The changes from 0–12 weeks and 12–24 weeks within the two groups separately will be assessed using paired t-test (autonomy score, age, gender and physical functioning) and/or Wilcoxon rank sum test for variables not fulfilling the normal distribution of residuals. Furthermore, bar charts will be made for delta values for the changes from 0–12 weeks and 12–24 weeks.

The analyses will be performed according to the intention-to-treat principle, and carried out using a regression model incorporating difference in changes (delta values), and controlling for baseline values, e.g. age and gender.

Furthermore, the content of the individual programmes and participants' activity wishes will be analysed using a thematic analysis.

Participants of this study represent a group of people who might be in a deficit of stimulation due to being physically frail and institutionalised. Therefore, changes in the control groups are expected.

Statistical significance is set at p < 0.05.

The statistics will be performed in cooperation with The Research Unit for Statistics, Faculty of Health Sciences at The University of Southern Denmark.

### Sample size estimate

Sample size estimations were initially based on the calculations done for the Nordic multicenter study. These estimations show that to obtain a power of at least 80% and a significance level of 5%, 60 participants in each group are sufficient. No previous studies have investigated the effect of individually tailored programmes on perceived autonomy and thus it was difficult to determine an appropriate sample size. Therefore it was decided to recruit the largest possible number of subjects and aim for at least 60 in each group.

## Discussion

Many studies concerning autonomy in nursing home settings focus either primarily on the staff perspective or evaluate how staff and residents perceive autonomy in pre-defined areas of daily life. A recent European study including five countries argues, in agreement with many other studies, that comparison of nurses' and residents' views on autonomy reflect different results [11]. However, studies focusing explicitly on how residents express their experience and perception of autonomy are scarce.

If the results of this study are promising, future efforts might be directed at developing guidelines for more effective approaches to ensure frail older institutionalised peoples' autonomy in daily life. The hypothesis is that these efforts will lead not only to experiences of autonomy but also to enhanced mobility, independence, participation and well-being.

## Competing interests

The authors declare that they have no competing interests.

## Authors' contributions

Both authors contributed to the design of the study and wrote the paper together. MA administered the study, was responsible for collecting the data and for performing the statistical analyses. LP provided guidance and supervision during the whole process. Both authors contributed significantly to the preparation of the paper, and read and approved the final version.

## Pre-publication history

The pre-publication history for this paper can be accessed here:



## References

[B1] WHO (1999). Ageing, Exploring the myths.

[B2] The Danish Technology Council, The Future Elderly, 2002 [Teknologirådet. Fremtidens ældre]. http://www.tekno.dk.

[B3] Forster A, Bailey J, Smith J, Young J, Green J, Burns E (2003). Rehabilitation for older people in long term care. The Cochrane Database of Systematic Reviews.

[B4] Avlund K (2004). Disability in old age – longitudinal population-based studies of the disablement process. Munksgaard Denmark.

[B5] Johannesen A, Petersen J, Avlund K (2004). Satisfaction in everyday life for frail 85-year-old adults: a Danish population study. Scand J Occup Ther.

[B6] Kane RA, Gamroth L, et al (1995). Autonomy and regulation in long-term care: Odd couple, an ambiguous relationship. Enhancing autonomy in long-term care.

[B7] Draper P (1996). Compromise, massive encouragement and forcing: a discussion of mechanisms used to limit the choices available to older adults in hospital. Journal of Clinical Nursing.

[B8] Rowels GD (1991). Beyond performance: Being in place as a component of occupational therapy. Am J Occup Ther.

[B9] Johannesen A (2004). A study of old people who in spite of frailty are coping successfully with changes in their daily life. Master of Science thesis.

[B10] Svidén G, Wikström BM, Hjortensjö-Norberg M (2002). Elderly persons' reflections on relocating to living at sheltered housing. Scand J Occup Ther.

[B11] Scott PA, Välimäki M, Leini-Kilpi H, Dassen T (2003). Autonomy, privacy and informed concent 3: Elderly care perspective. British Journal of Nursing.

[B12] Reinardy JR (1999). Autonomy, choice and decision-making: How nursing home social workers view their role. Social work in health care.

[B13] Kane RA, Caplan AL, Urv-Wong EK, Freeman IC, Aroskar MA, Finch M (1997). Everyday matters in lives of nursing home residents: wish for and perception of choice and control. J Am Geriatr Soc.

[B14] Shawler C, Rowels GD, High DM (2001). Analysis of key decision-making incidents in the life of a nursing home resident. The Gerontologist.

[B15] Legarth KH (2005). The most important activity and the reasons for that experience reported by a Danish population at age 75 years. Br J Occup Ther.

[B16] Lysack CL, Seipke HL (2002). Communicating the occupational self: a qualitative study of oldest-old American women. Scand J Occup Ther.

[B17] Davies S, Laker S, Ellis L (1997). Promoting autonomy and independence for older people within nursing practice: a literature review. Journal of Advanced Nursing.

[B18] Agich G (1993). Autonomy and long term care.

[B19] Jackson J, Zemcke R, Clark F (1996). Living a meaningful existence in old age. Occupational Science – The evolving discipline.

[B20] Johnson BD, Stone GL, Altmaier EM, Berdahl LD (1998). The relationship of demographic factors, locus of control and self-efficacy to successful nursing home adjustment. Gerontologist.

[B21] Bean J, Kiely DK, Leveille SG, Morris J (2002). Associating the onset of motor impairments with disability progression in nursing home residents. Am J Phys Med Rehabil.

[B22] Richardson J, Bedard M, Weaver B (2001). Changes in physical functioning in institutionalized older adults. Disabil Rehabil.

[B23] Ruuskanen JM, Parkatti T (1994). Physical activity and related factors among nursing home residents. J Am Geriatr Soc.

[B24] Przybylski BR, Dumont ED, Watkins ME, Warren SA, Beaulne AP, Lier DA (1996). Outcomes of enhanced physical and occupational therapy service in a nursing home setting. Arch Phys Med Rehabil.

[B25] Lazowski DA, Ecclestone NA, Myers AM, Paterson DH, Tudor-Locke C, Fitzgerald C, Jones G, Shima N, Cunningham DA (1999). A randomised outcome evaluation of group exercise programs in long-term care institutions. J Gerontol.

[B26] Baum EE, Jarjoura AE, Faur D, Rutchi G (2003). Effectiveness of a group exercise in a long-term care facility:a randomised pilot trial. J Am Med Dir Assoc.

[B27] Rydwik E, Frändin K, Akner G (2004). Effects of physical training on physical performance in institutionalised elderly patients (70+) with multiple diagnoses. Age and Ageing.

[B28] Haak M, Fänge A, Iwarsson S, Ivanoff DS (2007). Home as a significancation of independence and autonomy: experiences among very old Swedish people. Scand J Occup Ther.

[B29] Beck AM, Damkjær K, El Kohly K, Schroll M Plejetyngden på danske plejehjem [The nursing burden among older Danes living in nursing homes]. Ugeskrift for læger.

[B30] Leclerc G, Lefrançois R, Dubé M, Hébert R, Gaulin P (1998). Self-actualization concept: A content validation. Journal of Social Behavior and Personality.

[B31] Lefrançois R, Leclerc G, Dubé M, Hébert R, Gaulin P (1997). The development and validation of a self-report measure of self-actualization. Social Behavior and Personality: An International Journal.

[B32] Lefrançois R, Leclerc G, Dubé M, Hébert R, Gaulin P (1998). Reliability of a new measure of self-actualization. Psychological Reports.

[B33] Leclerc G, Lefrançois R, Dubé M, Hébert R, Gaulin P (1999). Criterion validity of a new measure of self-actualization. Psychological Reports.

